# Allergies in Urban Areas on the Rise: The Combined Effect of Air Pollution and Pollen

**DOI:** 10.3389/ijph.2021.1604022

**Published:** 2021-05-04

**Authors:** Amanda Gisler

**Affiliations:** University Children’s Hospital Basel, University of Basel, Basel, Switzerland

**Keywords:** air pollution, pollen, city, interaction, respiratory allergy, allergens

**The IJPH series “Young Researcher Editorial” is a training project of the Swiss School of Public Health**

Urban populations are expanding rapidly. UN estimates suggest that by 2050, two-thirds of the world’s population will live in cities [1]. In cities, green spaces are shrinking, but pollen allergies and allergic respiratory symptoms are still increasing in adults and children [2]. These allergies may be exacerbated by other factors like air pollution. In the European Union (EU), air pollution levels exceed World Health Organization (WHO) thresholds for 96% of the urban population [3, 4]. Air pollution alone has an adverse effect on respiratory health [5], and biological and chemical studies have shown that air pollution aggravates the allergenicity of pollen. Air pollutants increase the allergen content of pollen and damage its surface, releasing more allergens [6]. Air pollutants also make pollen more allergenic by changing its elemental composition, causing pollen to release more airborne sub-pollen particles and increasing total pollen count [6]. By this, air pollution combines with humidity and temperature to drive pollen count in the environment.

A growing number of researchers examine the air pollution-modified effect of pollen on human allergic respiratory symptoms and diseases [7, 8]. Experimental studies have provided evidence for this association [6]. But epidemiological studies on the interactive effect of pollen and air pollutants on allergies at ambient level report conflicting results [9]. Investigating the interaction between air pollution and pollen in real—world settings is challenging as it requires sufficient variation and correct classification of exposure data as well as adjustment for confounding factors such as e.g. temperature.

Epidemiological studies often analyze the association between pollen and respiratory health outcomes by using surrogates for pollen exposure like the normalized difference vegetation index, tree classification, season, or date of birth. While surrogate measures can be used to study the overall impact of vegetation on respiratory outcomes, an analysis of the effect of pollen and polluted pollen on allergic respiratory diseases should be based on species-specific pollen counts [8]. Ideally, researchers should combine explicit spatial and temporal pollen data with air pollution data estimated for each individual study participant. We need to collect more and better epidemiological evidence about interactions between air pollution and pollen, so we can understand how they drive allergies in urban space.

Further studies are needed to provide robust evidence that interactions between air pollution and pollen harm respiratory health, but we can already act to make city environments healthier by reducing air pollution levels and restrict the amount of allergenic pollen. Several European cities have already extended car-free zones (e.g. Brussels), introduced electric or hybrid taxis and buses (e.g. Dublin), and issue emission stickers that limit entrance into restricted zones to vehicles that meet emission limits (e.g. Germany). Furthermore, evidence-based green space planning can reduce the amount of polluted air trapped by tree canopies, and fosters to plant low-allergenic trees and grasses [10]. Reducing air pollution levels can prevent pollen from becoming more allergenic, while beneficial greening lowers exposure to inherently highly allergenic pollen.

To reduce the number of city dwellers who suffer from allergic respiratory diseases or symptoms caused by pollen and air pollutants, alone and in combination, we should promote interdisciplinary efforts that include biologists, epidemiologists, clinicians, landscape architects, policy makers and urban designers. These combined efforts are most likely to reveal the complex association between pollen and air pollution and to help us improve, devise and test future interventions to make the city environment a healthier place for people to live.
